# Efficacy of Bioengineered PD-L1 siRNA for Immunotherapy Against Non-Small Cell Lung Cancer Cells

**DOI:** 10.3390/ncrna12040026

**Published:** 2026-07-27

**Authors:** Neelu Batra, Mei-Juan Tu, Su Guan, Jonathan W. Riess, Ai-Ming Yu

**Affiliations:** 1 Department of Biochemistry and Molecular Medicine, UC Davis School of Medicine, Sacramento, CA 95817, USA; nbatra@health.ucdavis.edu (N.B.); suguan@health.ucdavis.edu (S.G.); 2 Division of Hematology and Oncology, UC Davis School of Medicine, Sacramento, CA 95817, USA; jwriess@health.ucdavis.edu

**Keywords:** bioengineered RNA, PD-L1, siRNA, immunotherapy, non-small cell lung cancer

## Abstract

**Background/Objectives**: Recent advances in immunotherapy have revolutionized cancer treatment, as exemplified by multiple monoclonal antibodies against programmed cell death protein 1 (PD-1) or programmed death-ligand 1 (PD-L1). Nevertheless, immunotherapeutic antibodies exhibit certain limitations, which drives the development of alternative approaches, such as small interfering RNA (siRNA)-based therapeutics. The aim of this study was to design and produce new biological PD-L1 siRNA (BioRNA/PD-L1-siRNA) molecules and further define their immunotherapeutic efficacy against non-small cell lung cancer (NSCLC) in vitro. **Methods**: A novel RNA molecular bioengineering platform was employed to produce new BioRNA/PD-L1-siRNA agents. The functions of BioRNA/PD-L1-siRNAs were determined by quantitative PCR, Western blot, immunofluorescence confocal imaging, flow cytometry, and PD-1/PD-L1 blockade assays in human NSCLC cells, alone and co-cultured with human peripheral blood mononuclear cells (PBMCs). **Results**: After heterologous overexpression and purification of five BioRNA molecules, one siRNA named BioRNA/PD-L1-siRNA-1 was identified as the most effective to selectively suppress human PD-L1 mRNA and protein levels in H460 and H1975 cells. Disruption of PD-1/PD-L1 interactions by BioRNA/PD-L1-siRNA-1 was further demonstrated via a PD-1/PD-L1 blockade bioassay. In addition, the immunomodulatory effectiveness of BioRNA/PD-L1-siRNA-1 was established in co-culture models, as indicated by the induction of T-cell and natural killer cell populations and an increase in specific cytokines and cytotoxic granules, and subsequent enhancement of apoptosis and greater inhibition of NSCLC cell viability. **Conclusions**: Overall, these findings demonstrate the potential of bioengineered PD-L1 siRNA entities for NSCLC immunotherapy.

## 1. Introduction

Cancer immunotherapy has achieved remarkable success by harnessing the host immune system to control malignant progression [1,2]. Central to this defense is immune surveillance, which recognizes and eliminates foreign invaders such as microorganisms and mutated tumor cells [3,4]. However, cancer cells can adapt to immune pressure through various escape mechanisms to evade attack by effector cells [5,6]. Immunotherapy aims to counteract these mechanisms and invigorate the immune response against malignancy [1,7]. One of the most common escape mechanisms is the upregulation of immune checkpoint molecules that suppress immune cell function, thereby fostering an immunosuppressive tumor microenvironment (TME) [7,8]. Notably, programmed death-ligand 1 (PD-L1) is a commonly upregulated immune checkpoint in cancer cells, which engages its receptor, programmed cell death protein 1 (PD-1) on the T cell surface to suppress T cell activation [9,10]. A number of immune checkpoint inhibitors (ICIs), such as monoclonal antibodies against PD-1/PD-L1, have been approved by the United States Food and Drug Administration (FDA) for the treatment of various cancers, including non-small cell lung cancer (NSCLC), advanced melanoma, and hepatocellular carcinoma [11,12,13,14]. Currently, eight anti-PD-1 antibodies (pembrolizumab, nivolumab, cemiplimab, dostarlimab, toripalimab, retifanlimab, tislelizumab, and penpulimab) and four anti-PD-L1 antibodies (atezolizumab, avelumab, durvalumab, and cosibelimab) have been approved for clinical use by the FDA, either as monotherapy or in combination with other treatments, and more antibody-based therapeutics are under investigation [12,15,16,17,18,19,20].

Lung cancer remains the leading cause of cancer-related deaths across all ethnic groups in the United States and worldwide, contributing to nearly 19% of all cancer deaths globally [21,22]. NSCLC is the most prevalent subtype of lung cancer, accounting for approximately 85% of all lung cancer cases [23,24]. Patient outcomes have significantly improved with the decline in smoking prevalence, increased detection of early-stage lung cancer, and the introduction of new treatment strategies such as targeted therapy and immunotherapy; however, lung cancer remains one of the cancers with the lowest 5-year relative survival rate at 28% [21,25,26]. While immunotherapies offer substantial survival benefits in NSCLC [27], besides causing certain immune-related adverse events, they share notable limitations with chemotherapies and targeted therapies, such as intrinsic and acquired resistance and restricted applicability to specific patient populations [28,29]. One of the most critical factors influencing these outcomes is the TME, a heterogeneous ecosystem comprising cancer cells, immune cells, stromal components, and signaling molecules that drives tumor progression, metastasis, and therapeutic response or resistance through reciprocal interactions among these components [30,31,32]. As a primary constituent of the TME, tumor-associated macrophages (TAMs) frequently polarize toward a pro-tumor M2 phenotype, thereby inducing immunosuppression by inhibiting cytotoxic T-cell activity and promoting malignancy via the secretion of anti-inflammatory cytokines and the upregulation of PD-L1 signaling [33,34]. TAMs have been implicated in angiogenesis, metastasis, and resistance to immune checkpoint blockade therapies in NSCLC [35,36]. To counteract these pro-tumor functions, therapeutic strategies targeting TAMs have emerged as promising approaches for improving immunotherapy outcomes, despite remaining critical challenges [34,37]. Therefore, novel therapeutic strategies are urgently needed to improve efficacy, overcome resistance, and prevent recurrence in NSCLC.

RNA interference (RNAi) with small interfering RNA (siRNA) or similar agents has emerged as a promising alternative to immune checkpoint antibodies [38,39]. As small double-stranded RNA molecules, siRNAs function by unwinding into single strands and binding specifically with complementary messenger RNA (mRNA) transcripts to silence targeted genes. This mechanism of action offers distinct advantages over small molecule therapeutics and antibody drugs, allowing for more selective targeting [40,41]. In addition, RNAi holds the potential to theoretically target any gene of interest. This flexibility might render a shorter research and development timeline and a broader therapeutic targeting for using RNAi agents, as compared to small molecules or antibodies, especially for genes that are otherwise difficult to target with traditional modalities [39,41]. Indeed, eight siRNA drugs, inclisiran, patisiran, lumasiran, nedosiran, givosiran, vutrisiran, fitusiran, and plozasiran, have been approved by the United States FDA for specific indications, and many others are in clinical trials (e.g., tivanisiran, teprasiran, and cosdosiran) [42,43,44], highlighting RNAi therapeutics as a novel class of medication. It is also noteworthy that one siRNA designed to selectively target PD-1, INTASYL™ compound PH-762, is undergoing a Phase 1 clinical study [45], underscoring the potential of RNAi-based immunotherapy.

Currently, siRNA-based research and therapeutics rely predominantly on RNA agents chemically synthesized in vitro, which incorporate extensive unnatural chemical modifications to enhance metabolic stability and improve immunocompatibility [41,46]. Recently, we have developed a novel robust RNA molecular bioengineering technology, based upon the tRNA-fused precursor microRNA (pre-miR; e.g., human hsa-pre-miR-34a) carriers [47,48], to enable in vivo heterologous overexpression of recombinant or bioengineered RNA (BioRNA) molecules with payload siRNAs or miRs that are readily purified for functional studies [49,50,51]. These BioRNA/siRNAs and BioRNA/miRs effectively complement conventional tools to delineate gene functions and explore new therapeutics.

In the present study, we aimed to target the PD-L1 immune checkpoint by using new siRNA entities designed and generated via the BioRNA platform technology, namely BioRNA/PD-L1-siRNA molecules. Among five molecules produced successfully, the one named BioRNA/PD-L1-siRNA-1 was identified to downregulate human PD-L1 effectively and selectively in NSCLC cells and robustly disrupt the PD-1/PD-L1 interactions in a PD-1/PD-L1 blockade bioassay. Additionally, the immunomodulatory effects of BioRNA/PD-L1-siRNA-1 were demonstrated in a human NSCLC cell-peripheral blood mononuclear cell (PBMC) co-culture system in vitro, as manifested by an increased release of multiple cytokines and induction of T cells and natural killer (NK) cells, leading to enhanced apoptosis and inhibition of NSCLC cell viability. These findings demonstrate the utility of this novel BioRNA/PD-L1-siRNA agent as a promising candidate for the research and development of NSCLC immunotherapy.

## 2. Results

### 2.1. Heterologous Overexpression and Purification of Novel Biologic PD-L1-siRNA Molecules

**Figure 1:**
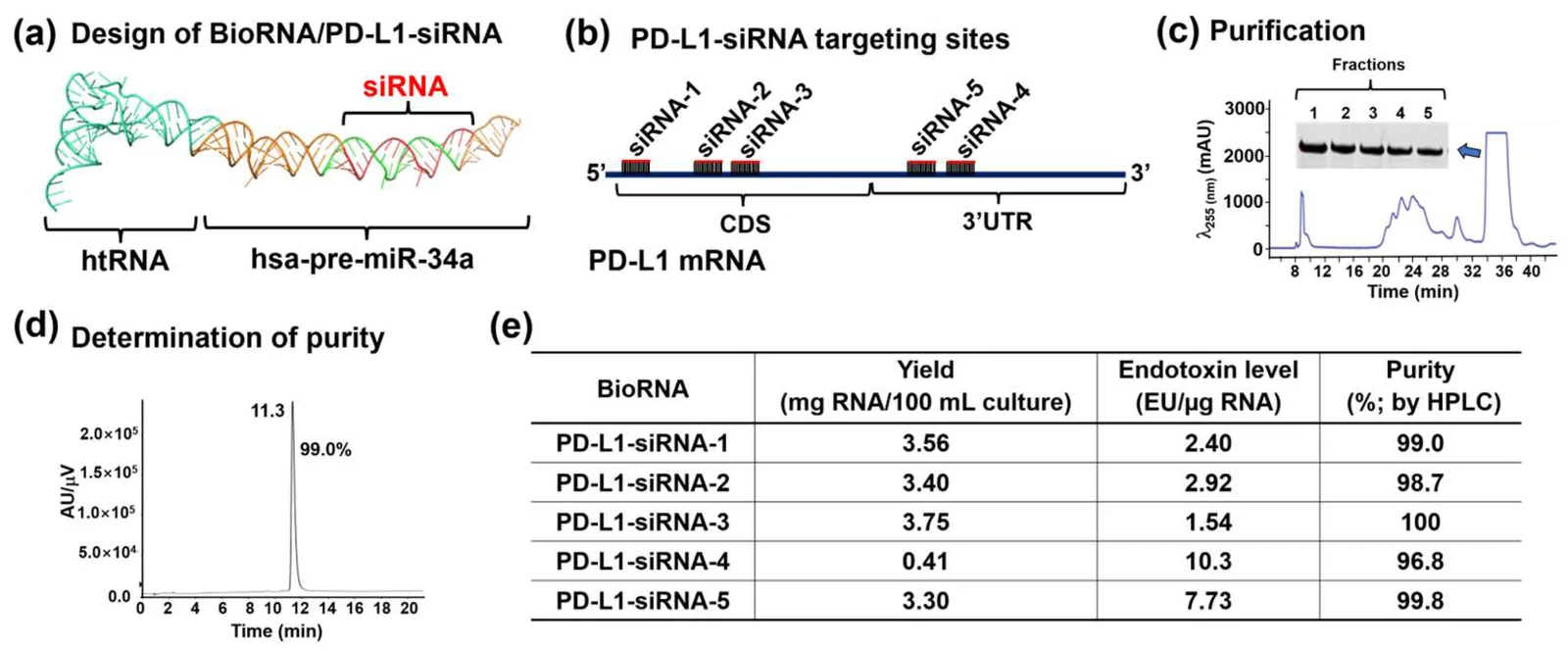
Bioengineering of novel PD-L1 siRNA molecules. (**a**) Schematic illustration of the human tRNA-fused hsa-pre-miR-34a carrier bearing target PD-L1 siRNA, namely BioRNA/PD-L1-siRNA. (**b**) Illustration of five PD-L1-siRNA molecules designed to act on different sites of the human *PD-L1/CD274* transcript; the siRNA-1 target site is specific to the human PD-L1 transcript, while the remaining sites are conserved between human and mouse PD-L1. (**c**) FPLC-UV trace during the purification of BioRNA/PD-L1-siRNA-1. The inset shows the urea-PAGE analysis of the collected BioRNA fractions. (**d**) Representative HPLC-UV trace showing the purity of the final BioRNA/PD-L1-siRNA-1 product. (**e**) Summary of the yield, purity, and endotoxin levels of individual BioRNA/PD-L1-siRNA molecules.

Five biological PD-L1 siRNAs targeting different regions of the human *PD-L1/CD274* transcript were designed using our optimized tRNA-fused hsa-pre-miR-34a carrier, as shown in Figure 1a. Among them, BioRNA/PD-L1-siRNA-1, -2, and -3 target the coding sequence (CDS), while BioRNA/PD-L1-siRNA-4 and -5 act on the 3′-untranslated region (3′U TR) (Figure 1b). Additionally, the BioRNA/PD-L1-siRNA-1 target site is specific to humans, whereas BioRNA/PD-L1-siRNAs 2–5 are conserved between human and mouse PD-L1. The four distinct BioRNA/PD-L1-siRNA (2–5) coding sequences were cloned into the pBSTNAV vector to generate individual BioRNA-expressing plasmids, which were subsequently verified through DNA sequencing. Plasmids were transformed into HST08 *E. coli* cells, and the overexpression of target BioRNA/PD-L1-siRNA was confirmed by urea-polyacrylamide gel electrophoresis (PAGE) analysis of bacterial total RNAs. Each BioRNA underwent anion-exchange fast protein liquid chromatography (FPLC) purification. Eluted fractions, monitored by UV absorbance (Figure 1c), were collected and verified as single RNA bands through urea-PAGE analysis (Figure 1c). Fractions were pooled, desalted, and concentrated to offer the final BioRNA product, with homogeneity determined by high-performance liquid chromatography (HPLC)-UV analysis (Figure 1d). Endotoxin levels were quantified using a Limulus amebocyte lysate (LAL) assay kit (Lonza Walkersville Inc., Walkersville, MD, USA). All FPLC-purified BioRNA/PD-L1-siRNAs, except BioRNA/PD-L1-siRNA-4, demonstrated high yield and homogeneity (Figure 1e). In addition, BioRNA/PD-L1-siRNA-1, -2, and -3 exhibited low endotoxin activity (<3 EU/μg RNA) (Figure 1e).

### 2.2. Variable PD-L1 Protein Basal Levels in NSCLC Cell Lines

**Figure 2:**
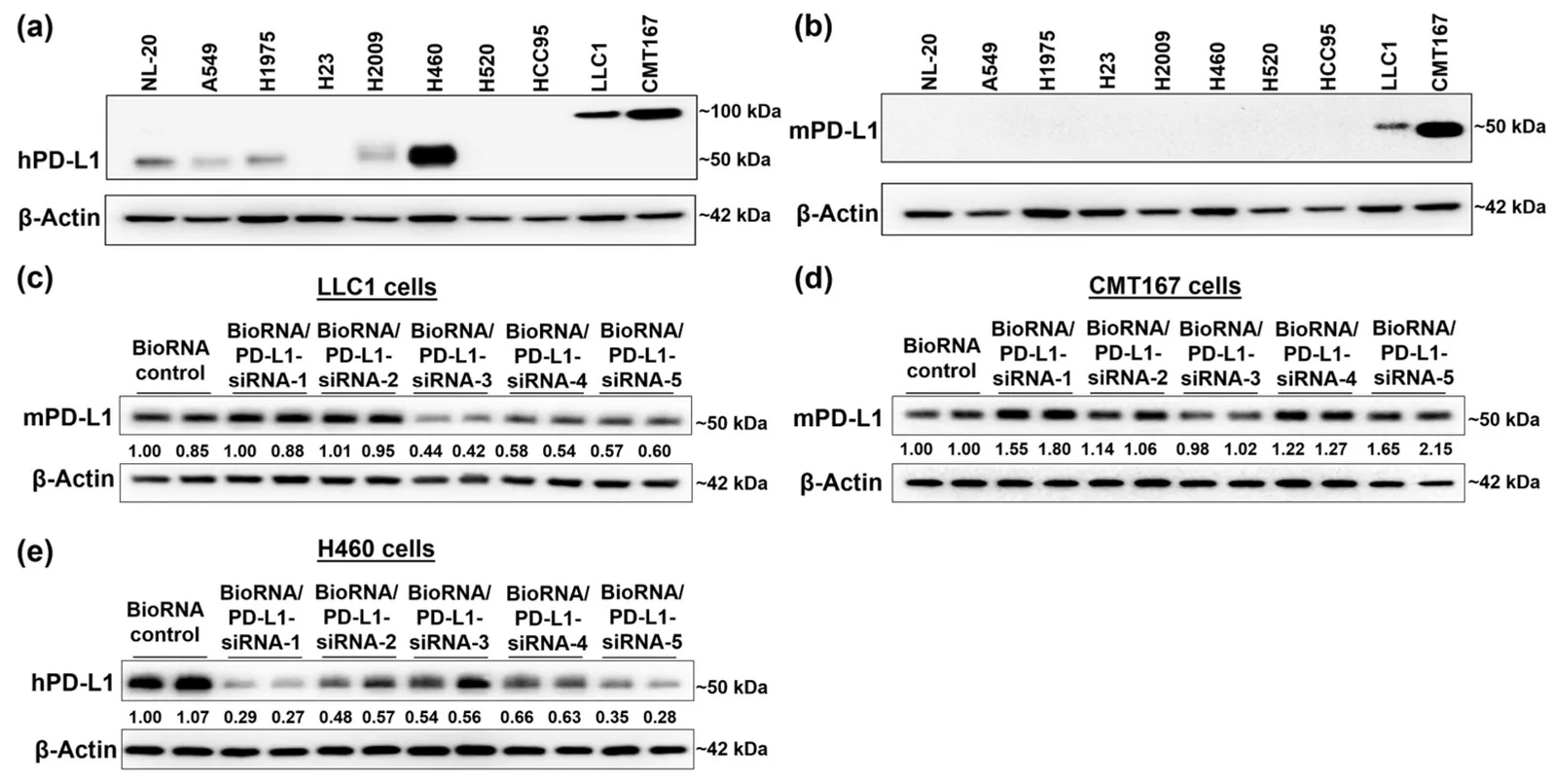
Comparison of the efficacy of five BioRNA/PD-L1-siRNAs to control PD-L1 protein outcomes in human and mouse lung cancer cell lines. (**a**,**b**)Immunoblot analyses of PD-L1 basal levels in multiple cell lines without any treatment, including one human normal lung epithelial (NL-20), seven human lung cancer (A549, H1975, H23, H2009, H460, H520, and HCC95), and two mouse lung cancer (LLC1 and CMT167) cell lines using human (**a**) and mouse (**b**) selective PD-L1 antibodies (hPD-L1 and mPD-L1, respectively). Consequently, LLC1 (**c**), CMT167 (**d**), and H460 (**e**) cells were chosen for immunoblot analyses of PD-L1 protein levels in response to 15 nM (for LLC1 and CMT167) or 5 nM (for H460) of individual BioRNA/PD-L1-siRNAs for 72 h. Experiments were conducted in biological duplicates. Target protein band density was normalized to the corresponding β-actin level and then to the control RNA group.

To identify proper cell models for functional study, the PD-L1 basal protein levels in seven human lung cancer cell lines, one human lung epithelial cell line, and two mouse lung cancer cell lines were determined by immunoblot analysis. As presented in Figure 2a, human NSCLC H460 cells exhibited a notably high human PD-L1 protein level at the expected size (~50 kDa); some cell lines (H1975, H2009) demonstrated a medium PD-L1 protein level, and others displayed minimal or undetectable PD-L1 expression. Meanwhile, a nominal level of human PD-L1 protein was observed in the normal lung epithelial NL-20 cells. To our surprise, the two mouse cell lines, LLC1 and CMT167, exhibited prominent, atypical immunoreactive bands at approximately double the expected size (~100 kDa), as detected by the antibody against human PD-L1 (PD-L1 (E1L3N^®^) XP^®^ Rabbit mAb #13684) (Figure 2a). By contrast, bands at the expected molecular weight (~50 kDa; Figure 2b) appeared in mouse LLC1 and CMT167 cells when analyzed with a mouse-specific antibody (PD-L1 (D4H1Z) Rabbit mAb #60475), revealing notably higher mouse PD-L1 protein level in CMT167 cells than in LLC1 cells.

### 2.3. Comparison of Five PD-L1-siRNA Activities to Reduce PD-L1 Protein Levels in Human and Mouse NSCLC Cells

We thus selected one human cell line (H460) and two mouse (LLC1 and CMT167) cell lines, which exhibit high to medium levels of PD-L1 protein, to examine the degree to which individual siRNAs could suppress human and mouse PD-L1 protein levels. The results showed BioRNA/PD-L1-siRNA-3 reduced mouse PD-L1 protein levels by about 55% in LLC1 cells but had minimal effect in CMT167 cells, while suppressing human PD-L1 protein levels by around 45% in H460 cells (Figure 2c–e). PD-L1-siRNA-4 and -5 showed similar results to siRNA-3. By contrast, PD-L1-siRNA-2 was not effective at suppressing mouse PD-L1 in either LLC1 or CMT167 cells, though displaying 50% efficacy against human PD-L1 in H460 cells. Overall, CMT167 cells exhibited resistance to all PD-L1-siRNAs, and BioRNA/PD-L1-siRNA-1 was revealed to be the most effective (~70%) to reduce human PD-L1 protein levels in human H460 cells (Figure 2e), with minimal effects on mouse PD-L1 (Figure 2c,d). Therefore, BioRNA/PD-L1-siRNA-1 was chosen for further investigation in human H460 and H1975 cells, which show high and moderate basal PD-L1 protein expression, respectively.

### 2.4. BioRNA/PD-L1-siRNA-1 Is Selectively Processed to Mature PD-L1-siRNA to Modulate PD-L1 Protein Levels in Human NSCLC Cells

**Figure 3:**
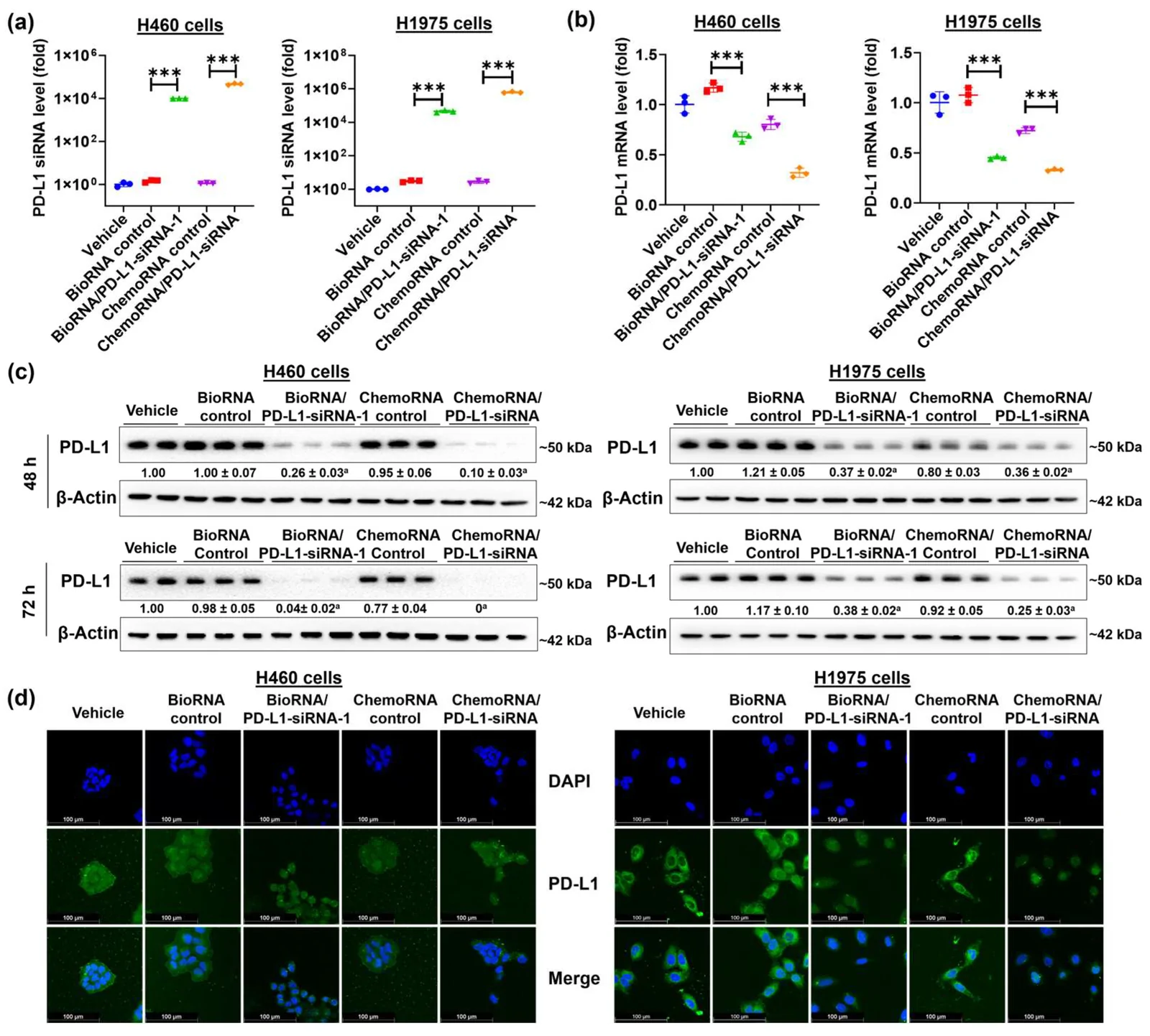
Effectiveness of BioRNA/PD-L1-siRNA-1 to regulate target gene expression in human NSCLC cells. (**a**) PD-L1 siRNA and (**b**) PD-L1 mRNA levels were remarkably elevated and decreased, respectively, in H460 and H1975 cells following transfection with 10 nM of BioRNA/PD-L1-siRNA-1 or its chemically synthesized counterpart, ChemoRNA/PD-L1-siRNA, for 72 h. siRNA and mRNA levels were determined by selective qPCR assays and normalized to U6 and 18S levels, respectively, and then to the vehicle control group. (**c**) PD-L1 protein levels were effectively downregulated by both the biological and chemical PD-L1 siRNAs to comparable degrees, as determined by immunoblot analysis. H460 and H1975 cells were treated with 10 nM of the PD-L1 siRNA agents for 48 and 72 h. Target protein band density values were normalized to the corresponding β-actin levels, and then to the vehicle control group. (**d**) Immunofluorescence analysis targeting PD-L1 further confirmed the efficacy of both siRNAs in reducing PD-L1 protein levels (green) localized to the cell membrane and cytoplasm. Cells were treated with 15 nM of the PD-L1 siRNA agents for 48 h. Values are the mean ± SD (n = 3/group, except for the vehicle group in immunoblots, which represent the mean of two samples randomly chosen from triplicate treatments). *** *p* < 0.001; ^a^ *p* < 0.05, compared to respective control RNA (one-way ANOVA with Bonferroni post hoc tests).

To delineate the release of PD-L1 siRNA from BioRNA/PD-L1-siRNA-1, we employed a selective reverse transcription (RT) quantitative real-time polymerase chain reaction (qPCR) analysis method as recently described [51] to quantitate PD-L1 siRNA in H460 and H1975 cells treated with either BioRNA/PD-L1-siRNA-1 or its commercial, chemically synthesized counterpart (ChemoRNA/PD-L1-siRNA). The results showed a remarkably high level of PD-L1-siRNA (Figure 3a) in the BioRNA/PD-L1-siRNA-1 treatment group that was comparable to the ChemoRNA/PD-L1-siRNA treatment group. Having established the successful processing of BioRNA/PD-L1-siRNA-1 into mature PD-L1 siRNA, its efficacy in regulating target PD-L1 mRNA and protein levels was further assessed by qPCR and immunoblot analyses, respectively. The data demonstrated a significant downregulation of both PD-L1 mRNA and protein levels by BioRNA/PD-L1-siRNA-1, to a degree (60–95% in protein) comparable to ChemoRNA/PD-L1-siRNA (Figure 3b,c). In addition, confocal imaging was also conducted to evaluate the impact of BioRNA/PD-L1-siRNA-1 on the PD-L1 protein outcome in H460 and H1975 cells, with ChemoRNA/PD-L1-siRNA included for comparison. As presented in Figure 3d, our data confirmed the downregulation of target PD-L1 by BioRNA/PD-L1 siRNA-1 compared to the corresponding controls. By contrast, the ChemoRNA control itself also reduced PD-L1 expression, rendering the downregulation by ChemoRNA/PD-L1-siRNA less remarkable, especially in H460 cells. Furthermore, imaging results indicated that PD-L1 siRNA treatment induced a shrunken morphology in both H460 and H1975 cells, with PD-L1 distinctly localized to the membrane and cytoplasm. Overall, these results demonstrate the selective release of PD-L1 siRNA from BioRNA/PD-L1-siRNA-1 and the effective downregulation of target PD-L1 at both mRNA and protein levels in H460 and H1975 cells.

### 2.5. BioRNA/PD-L1-siRNA-1 Disrupts the Interactions Between PD-1 and PD-L1

**Figure 4:**
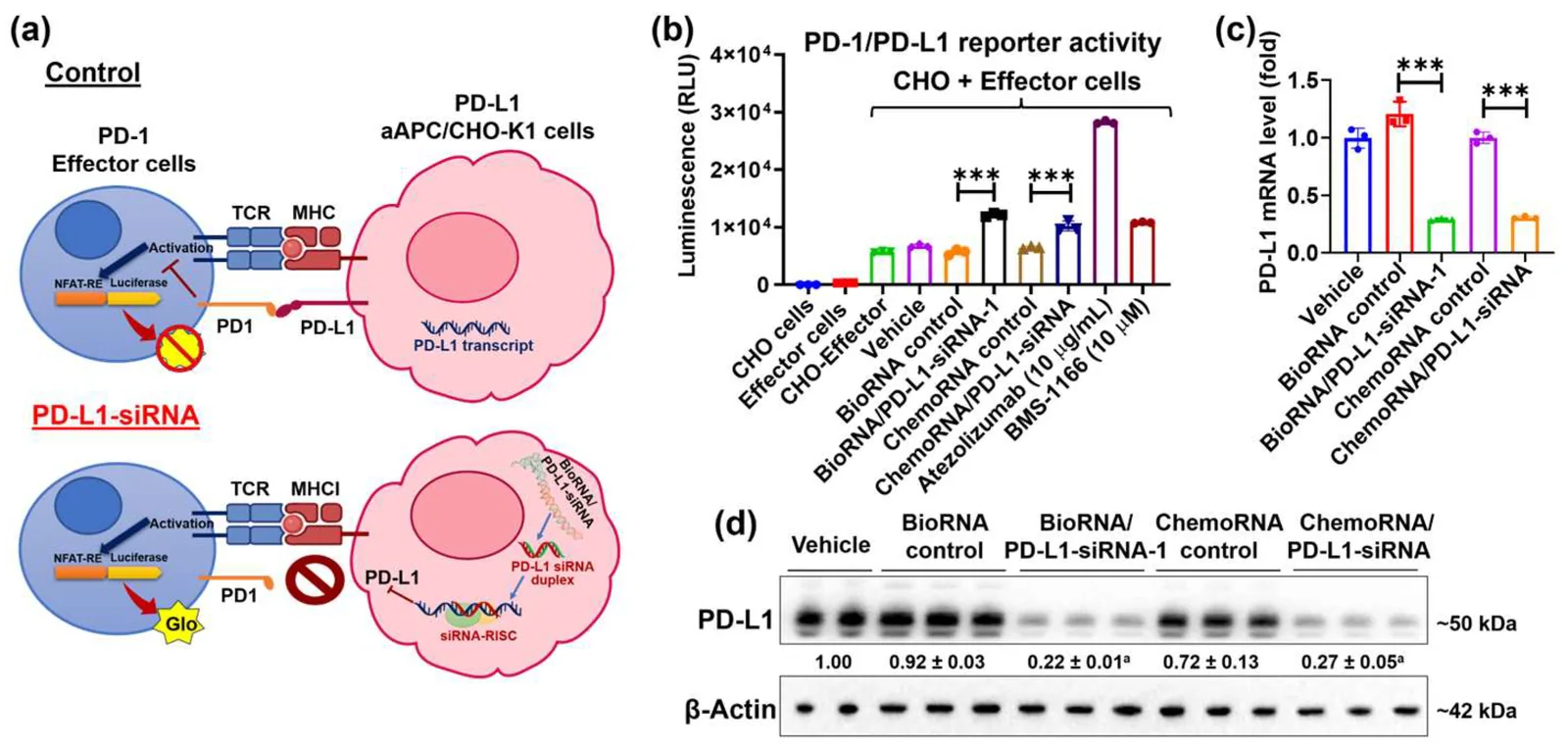
Impact of BioRNA/PD-L1-siRNA-1 on PD-1/PD-L1 immune checkpoint interactions. (**a**) Schematic illustration of the PD-1/PD-L1 blockade bioassay, in which disruption of the PD-1/PD-L1 interaction increases the reporter activity or luminescence signal. (**b**) Both BioRNA/PD-L1-siRNA-1 and ChemoRNA/PD-L1-siRNA effectively blocked the PD-1/PD-L1 interaction, as manifested by an increase in luminescence intensity, which was comparable to that of the small-molecule inhibitor BMS-1166 but lower than that of the PD-L1 antibody atezolizumab. The reduction in PD-L1 mRNA (**c**) and protein (**d**) levels by the siRNAs in the genetically engineered CHO cells was confirmed by qPCR and immunoblot analyses, respectively. Cells were transfected with 15 nM of the siRNA agents for 48 h. Values are the mean ± SD (n = 3/group, except for the vehicle group in immunoblots, which represents the mean of two samples randomly chosen from triplicate treatments). *** *p* < 0.001; ^a^ *p* < 0.05, compared to respective control RNA (one-way ANOVA with Bonferroni post hoc tests).

A PD-1/PD-L1 Blockade Bioassay, consisting of a PD-L1-expressing CHO cell line and a Jurkat T cell line stably expressing the nuclear factor of activated T-cells (NFAT) luciferase reporter and PD-1, was thus employed to define the effects of BioRNA/PD-L1-siRNA-1 on PD-1/PD-L1 interaction (Figure 4a). In the CHO cell and Jurkat T cell (effector cell) co-culture model, binding between PD-1 and PD-L1 leads to the suppression of NFAT activation, thereby reducing the luminescent signal, whereas disruption of this binding reactivates NFAT and restores luminescence (Figure 4a). Following treatment with BioRNA/PD-L1-siRNA-1, ChemoRNA/PD-L1-siRNA, or their respective controls, the monoclonal antibody (atezolizumab), or the small-molecule inhibitor BMS-1166, the CHO cells were co-incubated with Jurkat T cells to assess luminescence changes. The results showed that 15 nM of BioRNA/PD-L1-siRNA-1 significantly increased the transcriptional bioluminescent signal, to a degree comparable to that observed with 15 nM synthetic PD-L1-siRNA and 10 μM BMS-1166, but lower than that induced by 10 μg/mL atezolizumab (Figure 4b), demonstrating the strong PD-1/PD-L1 blockade effect induced by the biologic PD-L1 siRNA. In addition, the processing of BioRNA/PD-L1-siRNA-1 and the control of PD-L1 levels were also evaluated in the engineered CHO cells. The results demonstrated high levels of the siRNA antisense strand and the downregulation of PD-L1 mRNA and protein levels by the BioRNA/PD-L1-siRNA-1 agent, to an extent comparable to the ChemoRNA/PD-L1-siRNA (Figure 4c,d). Together, these findings suggest that BioRNA/PD-L1-siRNA-1 effectively disrupts PD-1/PD-L1 interactions via the knockdown of PD-L1 expression.

### 2.6. BioRNA/PD-L1-siRNA-1 Elevates the Inhibition of NSCLC Cell Viability via Induction of Immune Cells in a Human PBMC and NSCLC Cell Co-Culture System

**Figure 5:**
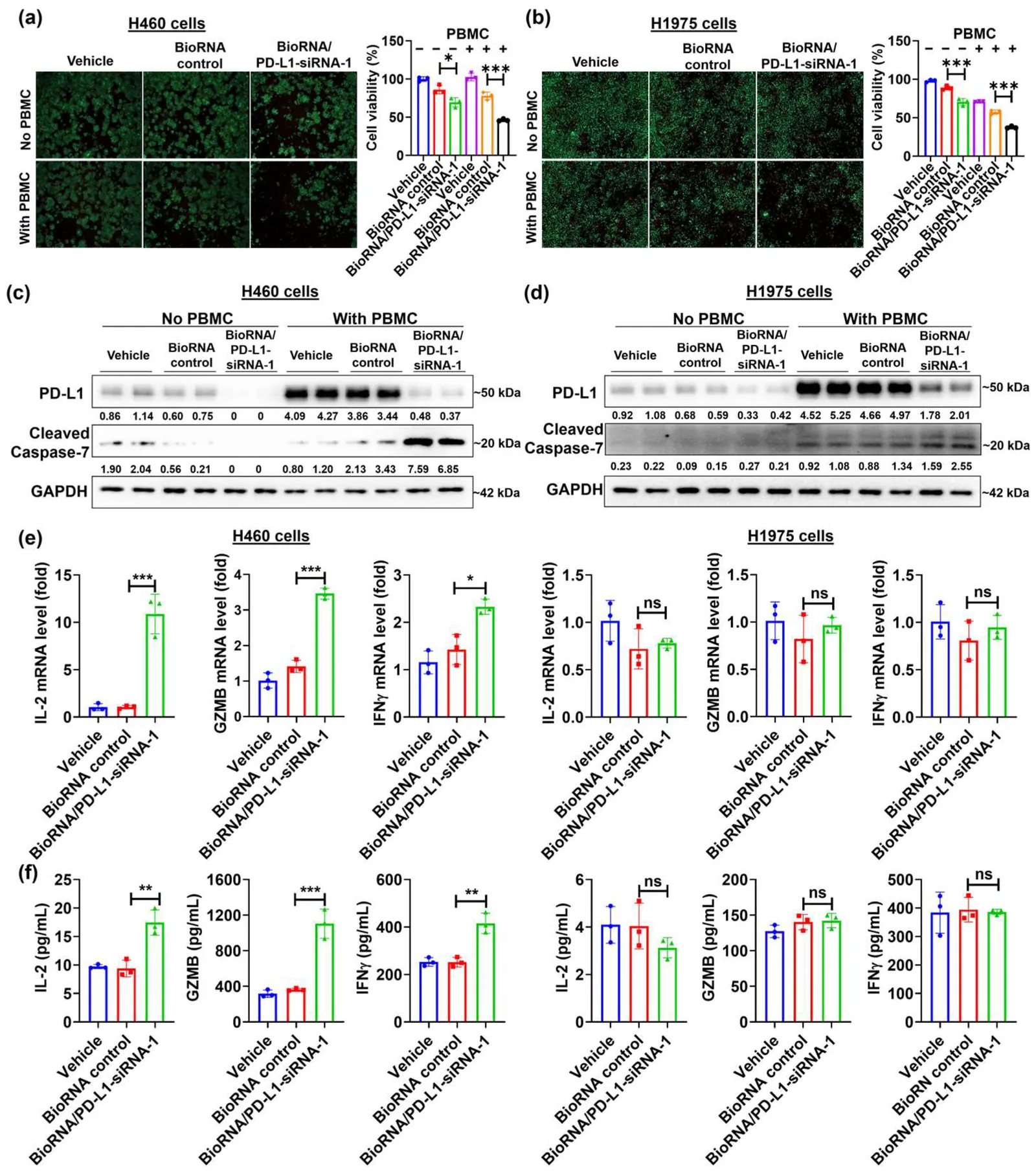
Efficacy of BioRNA/PD-L1-siRNA-1 in an NSCLC cell-PBMC co-culture environment. (**a**,**b**) BioRNA/PD-L1-siRNA exhibited the greatest anti-proliferative effect against NSCLC cells when co-cultured with human PBMCs, compared to the control or NSCLC cell-only groups. H460 and H1975 cells were transfected with 15 nM of RNA or vehicle for 24 h and then incubated with or without human PBMCs for an additional 48 h. Cell viability was determined by live/dead staining (left panel) and CellTiter-Glo (right panel) at the end of the incubation. Effects of BioRNA/PD-L1-siRNA-1 on PD-L1 and cleaved caspase-7 protein levels in H460 (**c**) and H1975 (**d**) cells with and without PBMCs after treatment, as determined by Western blot analyses. Target protein levels were normalized to the GAPDH levels in corresponding samples. PD-L1 was subsequently normalized to the vehicle group without PBMCs, and cleaved caspase-7 was normalized to the vehicle group with PBMCs. (**e**) Effects of BioRNA/PD-L1-siRNA on interleukin-2 (IL-2), granzyme B (GZMB), and interferon gamma (INFγ) mRNA in PBMCs after co-culture with H460 and H1975 cells, quantified by qPCR. (**f**) Impact of BioRNA/PD-L1-siRNA-1 on IL-2, GZMB, and INFγ levels in the co-culture media, as determined by Multiplexing LASER Bead Technology. Values are the mean ± SD (n = 3/group). * *p* < 0.05, ** *p* < 0.01, *** *p* < 0.001, and ns, not significant (one-way ANOVA with Bonferroni post hoc tests).

To determine whether BioRNA/PD-L1-siRNA-1 affects immune cell function by inhibiting PD-L1 expression, a co-culture model was established, in which BioRNA-treated H460 or H1975 cells were co-cultured with human PBMCs. Following 48 h of co-culture, cells were subjected to live/dead staining, and cell viability was further quantitated using a CellTiter-Glo assay. The results showed that treatment with BioRNA/PD-L1-siRNA-1 alone was effective in reducing the viability of both H460 and H1975 cells by approximately 30%, and co-incubation with PBMCs led to even greater degrees of inhibition (~55% and ~63%, respectively) (Figure 5a,b).

To evaluate the efficacy of BioRNA/PD-L1-siRNA-1 treatment in the co-culture environment at the molecular level, we examined PD-L1 protein levels in H460 and H1975 cells in the presence and absence of PBMCs. As expected, co-culture with PBMCs sharply elevated PD-L1 protein levels in both H460 and H1975 cells, which were notably suppressed by BioRNA/PD-L1-siRNA-1 (Figure 5c,d). Since apoptosis is a primary mechanism by which activated immune cells suppress tumor growth, the protein level of an apoptotic marker, cleaved caspase-7, was examined by immunoblot analysis. The results showed a marked increase in cleaved caspase-7 protein levels in BioRNA/PD-L1-siRNA-1-treated cells co-cultured with PBMCs compared to controls, indicating apoptosis as a cell death pathway in the co-culture system (Figure 5c,d). In contrast, cleaved caspase-7 was not elevated by PD-L1 siRNA in the cancer-cell-only group. This suggests that apoptosis is not the primary mechanism of PD-L1 siRNA-mediated NSCLC growth inhibition in the absence of immune cells.

**Figure 6:**
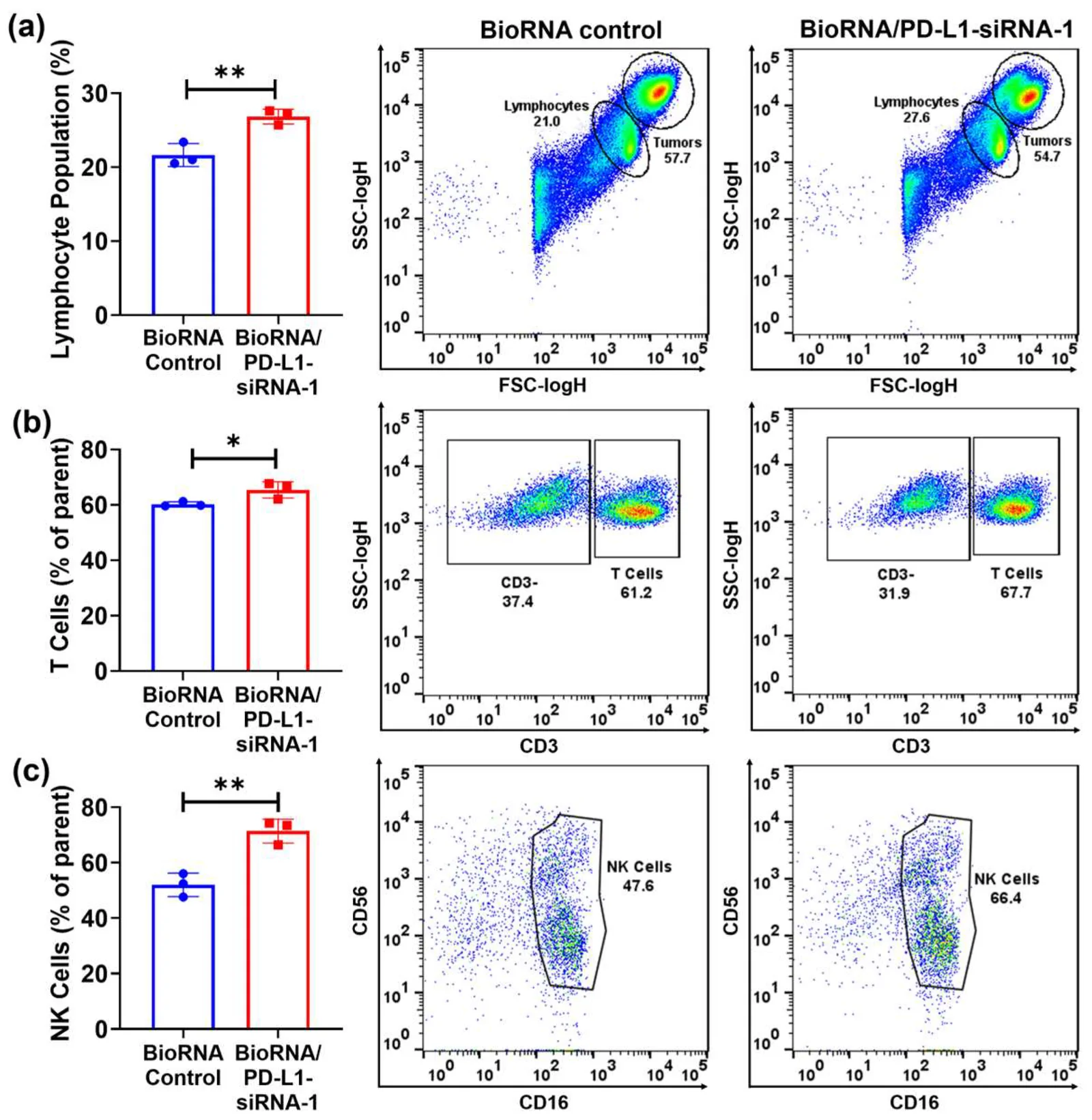
Modulation of lymphocyte populations by BioRNA/PD-L1-siRNA in the co-culture system. (**a**) Flow cytometry analysis of total lymphocyte population isolated from the co-cultured PBMCs and H460 cells in response to BioRNA/PD-L1-siRNA or control RNA treatment. (**b**) T cell and (**c**) NK cell populations among the PBMCs being isolated from the co-culture system. H460 cells were treated with 15 nM of BioRNA/PD-L1-siRNA-1 or control RNA for 24 h and then incubated with human PBMCs for an additional 48 h. Both the H460 cells and PBMCs were collected, incubated with representative biomarker antibodies, and subjected to flow cytometry analyses. Treatment with BioRNA/PD-L1-siRNA-1 resulted in an increase in both T cell and NK cell populations. Values are the mean ± SD (n = 3/group). * *p* < 0.05, ** *p* < 0.01 (unpaired, two-tailed Student’s *t*-test).

Furthermore, the mRNA levels of the immune cell activation markers, interleukin-2 (IL-2), granzyme B (GZMB), and interferon gamma (IFNγ), were measured in the isolated PBMCs from the co-culture system. A significant increase in all markers was found in PBMCs co-cultured with H460 cells treated with BioRNA/PD-L1-siRNA-1 compared to those treated with control RNA (*p* < 0.05) (Figure 5e). By contrast, no substantial change was observed in the H1975 co-culture groups (Figure 5e). Therefore, a panel of 20 common immune cell-secreted factors was further quantified. The results showed a notable increase in multiple cytokines, including IL-2, IFNγ, IL-10, and IL-13, as well as the cytotoxic effector proteins, such as granzyme A (GZMA), GZMB, and perforin, in the H460 co-culture group treated with BioRNA/PD-L1-siRNA-1 as compared with control RNA, whereas no statistically significant changes were observed in the H1975 co-culture groups (Figure 5f and Figure S1). Along with the elevated cytokines, the increased levels of GZMA, GZMB, and perforin typically indicate enhanced immune cell activation, especially involving cytotoxic T cells and NK cells, which play a pivotal role in targeting and destroying cancerous cells. Flow cytometry analyses were then performed to investigate the influence of BioRNA/PD-L1-siRNA-1 on lymphocyte, T-cell, and NK-cell populations. A consistent increase in the percentage of total lymphocytes, T cells and NK cells was observed in the H460 co-culture group treated with BioRNA/PD-L1-siRNA-1 as compared with control RNA, whereas no significant change was found in the H1975 co-culture groups (Figure 6 and Figure S2).

## 3. Discussion

Despite the demonstrated effectiveness of chemotherapies, whether used as a primary, adjuvant, or neoadjuvant treatment for the management of NSCLC, inherent or acquired chemoresistance remains an unavoidable challenge for many patients [52,53]. Targeted therapies (e.g., kinase inhibitors) and immunotherapies (e.g., anti-PD-1 and anti-PD-L1 antibodies) have revolutionized patient care and are now integrated into first-line treatment paradigms; nevertheless, such agents benefit only a subset of patients and can precipitate severe adverse events [54,55,56]. Overall, the existing treatment strategies are constrained by notable challenges, including their limited applicability across patient populations, variable response rates, and the presence of both primary and secondary resistance [30,57,58]. Therefore, the exploration and development of novel therapeutic approaches to combat NSCLC remain a critical clinical priority.

The advancement of RNAi technology, with its capacity to selectively silence disease-associated genes, has emerged as a promising alternative or complementary means to conventional therapies [42,43,44]. In comparison with small-molecule and protein-based therapeutics, RNAi provides a more specific, potentially less toxic, and versatile approach by directly targeting the genetic basis of diseases [41,42,59]. However, challenges related to unknown side effects arising from extensive artificial modifications of RNAi agents cannot be overlooked for broader clinical applications [41,42,46,59]. Therefore, the present study explored the function of PD-L1 siRNA agents produced and tolerated within live cells via a novel RNA molecular bioengineering technology [47,51], which authentically mimics the physicochemical and biological properties of endogenous RNA molecules.

Human and murine PD-L1 mRNA share approximately 76% identity (NM_014143.4 vs. NM_021893.3). However, their nucleotide-level conservation is highly fragmented, with no perfectly identical regions exceeding 20 nucleotides. To facilitate the study transition between human and mouse models, we designed four BioRNA/PD-L1-siRNA agents targeting the most highly conserved ‘islands’ within the CDS and 3′UTR of the transcripts. Following successful cloning and production, the novel BioRNAs and one human-specific PD-L1 siRNA (BioRNA/PD-L1-siRNA-1) described in our recent work [51] were purified to compare their efficacy in human and murine cell lines with high PD-L1 expression. However, none of the conserved siRNAs achieved remarkable PD-L1 downregulation in both species across the cell lines evaluated. The lack of cross-species efficacy may be attributed to several factors. Specifically, the thermodynamic stability and RISC binding affinity of these siRNAs likely differ between the two species due to variations in their sequences and target mRNA secondary structures. Currently, there are no reports of a single, cross-reactive siRNA sequence capable of effectively downregulating both human and murine PD-L1. These findings are consistent with previous reports indicating that while human and murine PD-L1 share similar protein structures, they exhibit distinct accessibility to anti-PD-L1 agents, including small molecules, peptides, and antibodies [60,61]. In line with this, the current study confirmed the superior efficacy and specificity of BioRNA/PD-L1-siRNA-1 in downregulating human PD-L1 expression. Consequently, two human NSCLC cell lines, H460 and H1975, with moderate to high PD-L1 expression levels were selected to investigate the therapeutic potential of this agent.

The results indicated that BioRNA/PD-L1-siRNA-1 was readily processed into PD-L1 siRNA to suppress PD-L1 expression, showing similar potency to its chemically synthesized counterparts. A subsequent PD-1/PD-L1 blockade assay demonstrated that BioRNA/PD-L1-siRNA-1 (15 nM) markedly activated immune effector cells by downregulating PD-L1 protein levels, with an effectiveness comparable to that of the small-molecule PD-L1 inhibitor BMS-1166 (10 μM), although lower than that of the PD-L1 antibody atezolizumab (10 μg/mL). BMS-1166 binds to PD-L1 through a hydrophobic pocket, inducing PD-L1 dimerization and blocking the PD-1/PD-L1 interaction; it is thus effective against both surface and intracellular PD-L1 [62], whereas antibody drugs predominantly interact with cell-surface PD-L1. In contrast to conventional therapeutics, siRNA drugs have the capacity to selectively downregulate PD-L1 expression at the post-transcriptional level, which may enhance therapeutic outcomes through the simultaneous disruption of tumor-intrinsic and immune-suppressive pathways [63]. The current study demonstrated that BioRNA/PD-L1-siRNA-1 exhibited comparable efficacy to the conventional drugs at a lower dose, highlighting its potential as a promising addition to current PD-1/PD-L1 targeting strategies. However, the efficient and tumor-specific delivery of a natural RNA agent represents a major barrier to clinical translation due to its inherent susceptibility to rapid nuclease degradation, limited cellular uptake within a highly heterogeneous and dynamic TME, and rapid renal clearance. Furthermore, potential off-target gene silencing and the requirement for sustained, selective knockdown underscore the complexity of in vivo application. To overcome these limitations, future studies should focus on establishing an optimal delivery platform with favorable stability and pharmacokinetic properties in vivo. Furthermore, a comprehensive comparison of the safety, efficacy, feasibility, and cost-effectiveness of these therapeutic modalities for in vivo treatment is warranted.

When co-cultured with human PBMCs, H460 cells treated with BioRNA/PD-L1-siRNA-1 exhibited substantial downregulation of PD-L1 protein, which triggered the induction of immune cells, particularly cytotoxic T cells and NK cells, and augmented the production of cytokines (e.g., IFNγ and IL-2) and cytotoxic effector molecules (e.g., GZMB and perforin) in the co-culture microenvironment. The elevated release of these effector molecules enhanced immune-driven cancer cell apoptosis, as evidenced by the upregulation of the apoptotic marker cleaved caspase-7. Intriguingly, while BioRNA/PD-L1-siRNA-1-treated H1975 cells in the human PBMC co-culture environment showed marked PD-L1 downregulation and increased levels of cleaved caspase-7, no evidence of immune cell activation was observed.

This differential response between H460 and H1975 cells to PD-L1 siRNA treatment in the co-culture environment may be attributed to variations in basal PD-L1 expression levels and underlying oncogenic signaling pathways. PD-L1 level is substantially lower in H1975 cells than in H460 cells (Figure 2a), suggesting that the PD-1/PD-L1 interaction may play a less critical role in H1975 survival; this lower expression likely explains the absence of detectable PBMC activation in the BioRNA-treated H1975 co-culture system. Beyond its role as a classic, well-characterized immune checkpoint, PD-L1 is also involved in various tumor-intrinsic signaling pathways, including those regulating cell survival, motility, and the epithelial–mesenchymal transition [63,64,65]. As such, genetic silencing of PD-L1 elicits a pronounced biological response in H460 cells via both immune-dependent and -independent pathways. Conversely, the lower PD-L1 expression observed in H1975 cells limits the impact of PD-L1 depletion, and antiproliferative effects are likely restricted to tumor-intrinsic pathways and the basal release of cytotoxic components from PBMCs under standard in vitro co-culture conditions.

Notably, H460 cells harbor a Kirsten rat sarcoma virus oncogene homolog (*KRAS*) mutation (Q61H), which has been shown to transcriptionally upregulate PD-L1 through the MAPK/ERK signaling pathway, thereby promoting immune evasion and enhancing cancer cell reliance on PD-L1 signaling [66,67,68,69]. Although most studies have not explored response variations across *KRAS* variants, a favorable correlation generally exists between *KRAS* mutations and PD-L1 expression; moreover, many mutation types showed a positive impact on overall survival of NSCLC patients treated with immunotherapy [68,70]. However, certain *KRAS* variants, such as the G12D mutation and co-mutations with serine threonine kinase 11 (*STK11*), or kelch-like ECH-associated protein 1 (*KEAP1*)/nuclear factor, erythroid 2 like 2 (*NFE2L2*), exert negative effects on immunotherapy efficacy [70]. By contrast, H1975 cells are driven by *EGFR* mutations (L858R/T790M) [71]. Although the exact role of EGFR mutations in driving PD-L1 expression remains controversial, clinical studies have shown that EGFR mutations are associated with low PD-L1 expression and poor responsiveness to PD-L1/PD-1 immunotherapy in NSCLC patients [72,73,74,75], which is consistent with our current findings. EGFR mutations can establish a “cold” or immunosuppressive TME through various pathways, including a lower tumor mutational burden, enhanced regulatory T cell infiltration alongside diminished tumor-infiltrating lymphocyte recruitment, and altered macrophage polarization, all of which contribute to resistance against ICI therapy [75,76]. Overall, the mechanisms behind the poor sensitivity of NSCLC cells to PD-1/PD-L1 blockade therapy are still not fully understood. To maximize the clinical benefits of NSCLC immunotherapy, a more granular investigation into the association between mutation subtypes, therapy response, and their underlying mechanisms is required. Taken together, our findings support the conclusion that the PD-L1 expression level is a critical determinant of cellular responsiveness to PD-L1-targeted siRNA approaches in NSCLC. Furthermore, specific gene mutation subtypes should be considered when stratifying subjects for optimized immunotherapy.

Beyond its effects on tumor cells, T cells, and NK cells, targeting the PD-1/PD-L1 axis can also modulate the broader TME, encompassing the regulation of TAM polarization. M2-type TAMs have been reported to drive drug resistance in ICI immunotherapy through multiple mechanisms, including the upregulation of PD-L1 expression on both TAMs and cancer cells [77]. Concurrently, blockade of the PD-1/PD-L1 axis and the subsequent shift in cytokine release may facilitate macrophage reprogramming toward an anti-tumor phenotype, thereby enhancing immune-mediated tumor clearance [78,79]. Therefore, future studies investigating how PD-L1-siRNA-1 modulates macrophage signaling and promotes broader TME remodeling, as well as the bidirectional interactions between the TME and cancer cells, may provide valuable insights into its therapeutic potential in NSCLC immunotherapy.

In conclusion, this study demonstrates the successful production of new biologic PD-L1 siRNAs and the effectiveness of BioRNA/PD-L1-siRNA-1 in suppressing PD-L1 expression in human NSCLC cells. By blocking PD-1/PD-L1 interactions, PD-L1-siRNA-1 activates immune cells, leading to greater apoptosis and enhanced inhibition of the viability of NSCLC cells with relatively high PD-L1 levels in the co-culture model system in vitro. These findings support the potential of the novel biologic PD-L1-siRNA-1 as a promising candidate for NSCLC immunotherapy.

## 4. Materials and Methods

### 4.1. Chemicals and Materials

Dulbecco’s Modified Eagle Medium (DMEM), RPMI 1640 medium, Ham’s F12 medium, 0.05% Trypsin-EDTA, fetal bovine serum (FBS), Phosphate-buffered saline (PBS); Lipofectamine 3000 transfection reagent, radioimmunoprecipitation assay (RIPA) buffer, and BCA protein assay kit were purchased from Thermo Fisher Scientific (Waltham, MA, USA). Protease inhibitor cocktail and Trizol reagent were bought from Sigma-Aldrich (St. Louis, MO, USA). PD-1/PD-L1 Blockade Bioassay kit (Cat# J1250) was purchased from Promega (Madison, WI, USA). Clarity Western ECL substrate, Blotting-grade Blocker, PVDF membrane, TGX Stain-Free Fast Cast Acrylamide kit (10%), and Universal SYBR^®^ Green Supermix were purchased from Bio-Rad (Hercules, CA, USA). Direct-Zol RNA miniprep kit was purchased from Zymo Research (Irvine, CA, USA). Commercial, chemically synthesized PD-L1 siRNA (ChemoRNA PD-L1 siRNA) (Cat# 10620312), with the same sequence as the PD-L1-siRNA-1 payload expressed from the BioRNA carrier, and the corresponding ChemoRNA control were obtained from Thermo Fisher Scientific. BMS-1166 (Cat# NC1778742) was bought from MedChemExpress (Monmouth Junction, NJ, USA). Atezolizumab was supplied by Genentech (South San Francisco, CA, USA). All primers, including those for the mRNA of GZMB, IL2, IFNγ, and PD-L1, and primers for mature PD-L1 siRNA were purchased from Integrated DNA Technologies (IDT; Coralville, IA, USA). All other chemicals and organic solvents of analytical grade were purchased from Thermo Fisher Scientific, VWR (Radnor, PA, USA), or Sigma-Aldrich.

### 4.2. Cell Culture

Human lung cancer cell lines H1975, H460, A549, H23, H2009, and H520, lung epithelial cell line NL20, and mouse lung cancer cell line LLC1 were purchased from American Type Culture Collection (Manassas, VA, USA). Human lung cancer cell line HCC95 and mouse lung cancer cell line CMT167 were purchased from Sigma-Aldrich. All cell lines were tested and confirmed to be mycoplasma free. Human PBMCs were purchased from Lonza. The cell lines and the PBMCs were maintained in either RPMI 1640 medium or DMEM supplemented with 10% fetal bovine serum and grown at 37 °C in a humidified incubator with 5% CO_2_. PD-L1 aAPC/CHO-K1 cells obtained from the PD-1/PD-L1 Blockade Bioassay kit were cultured in Ham’s F12 medium with 10% FBS, 200 μg/mL hygromycin B, and 250 μg/mL G418 sulfate solution. PD1 effector cells from the same kit were maintained in L-glutamine-containing RPMI 1640 medium, supplemented with 10% FBS, 200 μg/mL hygromycin B, 500 μg/mL G418 sulfate solution, 1 mM sodium pyruvate, and 0.1 mM MEM nonessential amino acids, as stated in the manufacturer’s protocol.

### 4.3. Design, Cloning, Overexpression and Purification of BioRNA/PD-L1-siRNA Molecules

Five 22-nucleotide sequences complementary to three different sites within CDS and two sites within the 3′UTR of the human *PD-L1* (or *CD274*) transcript were selected as the antisense sequences (Supplementary Table S1) of novel PD-L1 siRNAs. Among them, siRNA-1 specifically targets human PD-L1, while all others are designed to target sites conserved in human and mouse PD-L1. The cloning and production of BioRNA/PD-L1-siRNA-1 has been described in our recent study [51]. The novel BioRNA/PD-L1-siRNA-2 to -5 were designed following the same strategy [47,48,51], by inserting each target siRNA antisense sequence into the tRNA-fused pre-miRNA-34a carrier. Briefly, the coding sequences of the BioRNA/PD-L1-siRNAs (Supplementary Table S1) were obtained by PCR amplification using specific cloning primers (Supplementary Table S2) and subsequently inserted into the pBSTNAV vector via an In-Fusion cloning kit (Takara Bio USA; Mountain View, CA, USA), resulting in the construction of BioRNA expression plasmids. Sequence-verified plasmids were subsequently transformed into HST08 *E. coli* competent cells, and BioRNA accumulation levels were determined by denaturing urea-PAGE analysis of the total RNA extracted from bacteria. BioRNAs were then purified from the total RNA using an anion exchange FPLC method [48,51]. Target BioRNA fractions were collected and verified by urea-PAGE analysis, and the pure fractions were subsequently pooled, desalted, and concentrated to obtain the final products. BioRNA purity was quantified using HPLC, and endotoxin levels were measured via an LAL assay kit (Figure 1).

### 4.4. RNA Isolation, RT, and qPCR Analysis

H460 and H1975 cells were seeded in 6-well plates at a density of 5 × 10^5^ cells/well and incubated overnight for attachment, and then transfected with 10 nM of RNA formulated with Lipofectamine 3000 for 48 h. The RNA extraction and RT qPCR analysis were performed as previously described [51]. In brief, total RNA was isolated from the treated cells using a Direct-Zol RNA Miniprep Kit and quantified. cDNA was then synthesized from 300–500 ng total RNA using NxGen M-MuLV reverse transcriptase (Lucigen, Middleton, WI, USA) with random hexamer primers (Thermo Fisher Scientific) or siRNA-specific stem-loop primers. Real-time qPCR was performed on a CFX96 Touch real-time PCR system (Bio-Rad Laboratories, Hercules, CA, USA) using iTaq Universal SYBR Green Supermix (Bio-Rad Laboratories) with target gene-specific primers reported previously (Supplementary Table S3) [51,78,80], following the manufacturer’s instructions. Small RNA and mRNA levels were normalized to *U6* and *18S*, respectively, in the corresponding samples and then compared to the control group using the 2^−ΔΔC^_T_ method, where ΔC_T_ = C_T_ (target gene) − C_T_ (*U6*/*18S*), and ΔΔC_T_ = ΔC_T_ (treatment group) − ΔC_T_ (control group).

### 4.5. Protein Isolation and Immunoblotting

Untreated lung cancer and normal cell lines (for the determination of basal PD-L1 protein levels), or RNA- or vehicle-treated cells (5–15 nM for 48 h or 72 h; for the determination of siRNA efficacy) were harvested and lysed using RIPA buffer supplemented with a protease inhibitor cocktail for immunoblot analysis. A total of 20–30 µg of protein per sample was separated on a 10% SDS-PAGE gel, transferred to a polyvinylidene difluoride (PVDF) membrane, and blocked with 5% milk for 2 h. Membranes were then incubated overnight at 4 °C with specific primary antibodies against the target proteins human PD-L1 (Cell Signaling Technology, CST; Danvers, MA, USA; 13684S, 1:1000), mouse PD-L1 (CST; 60475S, 1:1000), or cleaved caspase-7 (CST; 9491S, 1:1000); or against the housekeeping proteins GAPDH (CST; 2118L, 1:3000) or β-actin (Sigma-Aldrich, A5441, 1:5000). After three 10 min washes, membranes were incubated with either anti-mouse (CST; 7076S, 1:3000) or anti-rabbit (Jackson ImmunoResearch, West Grove, PA, USA; 111-035-003, 1:10,000) HRP-conjugated secondary antibodies for 2 h at room temperature, followed by another round of three 10 min washes. Protein bands were developed using the Clarity Western ECL substrate (Bio-Rad Laboratories) as per the manufacturer’s protocol and imaged using the ChemiDoc MP Imaging System (Bio-Rad Laboratories). The intensity of the target protein bands was normalized to GAPDH or β-actin using Image Lab software (6.1) and then to the control group.

### 4.6. Immunofluorescence with Confocal Microscopy

H460 or H1975 cells were seeded in 8-well chamber slides at a density of 10,000 cells/well and incubated overnight for attachment. The following day, cells were transfected with 15 nM of BioRNA/PD-L1-siRNA-1, ChemoRNA/PD-L1-siRNA, or their respective control RNAs, and incubated for 48 h. Cells were then washed twice with ice-cold PBS and fixed with cold 4% paraformaldehyde for 20 min at 4 °C. Next, the cells were washed thrice for 10 min each with PBS and blocked with 3% BSA for 30–40 min at room temperature. Subsequently, the fixed cells were incubated overnight at 4 °C with an Alexa Fluor^®^ 488-conjugated PD-L1 (Extracellular Domain Specific, D8T4X) antibody (CST; 86744S), diluted 1:200 in 3% BSA. This was followed by three PBS washes and a 5 min incubation with 1 µg/mL 4′,6-diamidino-2-phenylindole (DAPI) to stain the nuclei. Following a 10 min wash with PBS, 20 µL of VECTASHIELD Antifade mounting medium was added to each well and then slides were sealed. Images were captured using a Leica Stellaris 5 confocal microscope system (Leica Microsystems Inc., Deerfield, IL, USA), integrated with a Leica Dmi8 Inverted Microscope and controlled through the Leica Application Suite software (v 4.4.0.24861).

### 4.7. PD-1/PD-L1 Blockade Bioassay

To evaluate the activity of the PD-L1 siRNA in the induction of immune response, PD-1/PD-L1 blockade bioassay was conducted on the siRNA-treated PD-L1 aAPC/CHO-K1 cells according to the manufacturer’s protocol and as previously described [51]. In brief, PD-L1 aAPC/CHO-K1 cells were seeded at a density of 10,000 cells/well in 200 μL RPMI medium in 96-well plates and incubated overnight. The cells were then transfected with 15 nM of BioRNA control, BioRNA/PD-L1-siRNA-1, ChemoRNA control, ChemoRNA/PD-L1-siRNA, or vehicle control. In addition, the cells were also treated with an FDA-approved PD-L1 monoclonal antibody, atezolizumab (10 μg/mL), or a small-molecule inhibitor BMS-1166 (10 μM) as positive controls. After a 48 h incubation, PD-1 effector cells (50,000 cells/well) were added in 80 μL of assay buffer (99% RPMI-1640 supplemented with L-glutamine and 1% FBS) and incubated for another 6 h. Bio-Glo™ Reagent (80 μL) was then added to each well, and the plate was incubated at room temperature for approximately 30 min. The medium was subsequently transferred to a white opaque 96-well plate, and luminescence was measured using a microplate reader (SpectraMax^®^ M3, Molecular Devices, Sunnyvale, CA, USA).

### 4.8. Co-Culture of Human NSCLC Cells with PBMCs

H460 and H1975 cells were seeded in 6-well plates (5 × 10^5^ cells/well) and 96-well plates (5000 cells/well) and incubated overnight for attachment. Meanwhile, frozen PBMCs were thawed and allowed to recover for two days. Cancer cells were then transfected with 15 nM of control RNA, BioRNA/PD-L1-siRNA-1, or vehicle control. After 24 h, the cancer cell culture medium was replaced with PBMC suspension at a density of 2 × 10^6^ cells/well for 6-well plates and 2 × 10^4^/well for 96-well plates and incubated for another 48 h. Following co-incubation, the cells in 96-well plates were subjected to live/dead staining (LIVE/DEAD™ Viability/Cytotoxicity Kit, for mammalian cells, Thermo Fisher Scientific) according to the manufacturer’s instructions and then imaged using an ImageXpress Pico imaging system (Molecular Devices, San Jose, CA, USA). Another set of cells under the same treatment condition was lysed with the CellTiter-Glo assay kit (Promega) following the manufacturer’s instructions to evaluate the cell viability. For the 6-well plates, PBMCs in the medium were harvested for qPCR analyses, the culture media were collected for cytokine quantification, and the cancer cells were harvested for immunoblot analyses. A separate set of cells was also treated under the aforementioned conditions in 6-well plates. After co-incubation, the PBMC-containing media were collected, and the attached cells were harvested by trypsinization, neutralization, and centrifugation. Then, the cell pellets were mixed with the collected cell suspension for flow cytometry analyses.

### 4.9. Flow Cytometry Analyses of the Lymphocyte, NK-Cell and T-Cell Populations

Flow cytometry analysis was performed at the Immune Modeling, Analysis and Diagnostics Shared Resource at the UC Davis Comprehensive Cancer Center. In brief, the mixed cell suspension was centrifuged at 350× *g* for 5 min and then the cells were resuspended and incubated with 100 μL of pre-mixed FACS buffer supplemented with BD Horizon Brilliant Stain buffer and BD Pharmingen Human BD Fc Block (BD Biosciences, San Jose, CA, USA), and Zombie NIR live/dead dye (BioLegend, San Diego, CA, USA) for 15 min at room temperature in the dark. The samples were then centrifuged and resuspended in a cocktail of fluorochrome-conjugated antibodies that selectively recognize human CD3 (FITC) and CD16 (BV805) (BD Biosciences), and CD56 (APC, Miltenyi Biotec, Gaithersburg, MD, USA) in FACS buffer. Following incubation with the antibodies at 4 °C for 30 min under light protection, the cells were washed in FACS buffer and fixed with 4% PFA for 15 min at 4 °C. Afterward, the cells were washed once and resuspended with 200 µL of FACS buffer and then injected to a Stratedigm S1000 EON Spectra flow cytometer (Stratedigm, Inc.; San Jose, CA, USA). The data were analyzed using FlowJo software (v10.10.0).

### 4.10. Quantification of Cytokine Levels

A Human Focused 15-Plex Discovery Assay^®^ and a Human CD8+ 5-Plex Discovery Assay^®^ (Eve Technologies Corporation, Calgary, AB, Canada) (https://www.evetechnologies.com/discovery-assay (accessed on 3 April 2026)) were used to quantify a panel of 15 human cytokines and 5 additional immune cell factors within the co-culture media described above.

### 4.11. Statistical Analysis

Comparisons between different treatment groups were performed using one-way ANOVA followed by Bonferroni post hoc tests or Student’s *t*-test (GraphPad Prism 11). The difference was deemed statistically significant if the *p* value was less than 0.05 (*p* < 0.05). Values are presented as the mean ± SD.

## 5. Conclusions

The present study designed and produced five new biologic PD-L1 siRNA (BioRNA/PD-L1-siRNA) agents using a novel RNA biotechnology. One BioRNA/PD-L1-siRNA molecule effectively suppressed PD-L1 expression and repressed the PD-1/PD-L1 interactions, leading to greater inhibition of human lung cancer cell growth as co-cultured with human PBMCs. The results demonstrate the promise of bioengineered PD-L1 siRNA as novel entity for immunotherapy.

## Data Availability

The original data presented in the study are openly available in Zenodo at DOI: 10.5281/zenodo.19383679.

## Supplementary Materials

The following supporting information can be downloaded at: https://www.mdpi.com/article/10.3390/ncrna12040026/s1, Figure S1: Impact of BioRNA/PD-L1-siRNA on the levels of a panel of cytokines in a human NSCLC carcinoma cell-PBMC co-culture system; Figure S2: Impact of BioRNA/PD-L1-siRNA on the levels of lymphocytes in the human NSCLC cell-PBMC co-culture system; Table S1: Primary sequences of individual BioRNA/PD-L1-siRNA molecules; Table S2: The sequences of primers utilized for the construction of BioRNA expression plasmids; Table S3: The sequences of primers used for RT-qPCR analyses in this study.

## Abbreviations

The following abbreviations are used in this manuscript:

BioRNA	Bioengineered RNA
CDS	Coding sequence
ChemoRNA	Chemically engineered RNA
DAPI	4ʹ,6-diamidino-2-phenylindole
DMEM	Dulbecco’s Modified Eagle Medium
EGFR	Epidermal growth factor receptor
FDA	Food and Drug Administration
FPLC	Fast protein liquid chromatography
GZMA	Granzyme A
GZMB	Granzyme B
HPLC	High-performance liquid chromatography
ICI	Immune checkpoint inhibitor
IL-2	Interleukin-2
INFγ	Interferon gamma
KEAP1	Kelch-like ECH-associated protein 1
KRAS	Kirsten rat sarcoma virus oncogene homolog
LAL	Limulus amebocyte lysate
mRNA	Messenger RNA
NFAT	Nuclear factor of activated T-cells
NFE2L2	Nuclear factor, erythroid 2 like 2
NK cell	Natural killer cell
NSCLC	Non-small cell lung cancer
PAGE	Polyacrylamide gel electrophoresis
PBMC	Peripheral blood mononuclear cell
PCR	Polymerase chain reaction
PD-1	Programmed cell death protein 1
PD-L1	Programmed death-ligand 1
qPCR	Quantitative real-time PCR
RT	Reverse transcription
RIPA	Radioimmunoprecipitation assay
RNAi	RNA interference
siRNA	Small interfering RNA
sFAS	Soluble FAS
STK11	Serine threonine kinase 11
TME	Tumor microenvironment
TAM	tumor-associated macrophage
3′UTR	3′-untranslated region
