# Supplementary material for: Efficacy of Bioengineered PD-L1 siRNA for Immunotherapy Against Non-Small Cell Lung Cancer Cells
Source: Noncoding RNA. 2026 Jul 27;12(4):26. doi: 10.3390/ncrna12040026 (PMC13516847; doi:10.3390/ncrna12040026)
Supplement: Supplementary file 1 [file ncrna-12-00026-s001.zip › ncrna-4269747-supplementary.pdf]

Supplementary materials to

Efficacy of Bioengineered PD-L1 siRNA for Immunotherapy Against Non-Small Cell Lung Cancer Cells

Neelu Batra [1,†], Mei-Juan Tu [1,*,†], Su Guan [1], Jonathan W. Riess [2] and Ai-Ming Yu [1,*]

[1] Department of Biochemistry and Molecular Medicine, UC Davis School of Medicine, Sacramento, CA 95817, USA; nbatra@health.ucdavis.edu (N.B.); suguan@health.ucdavis.edu (S.G.)
[2] Division of Hematology and Oncology, UC Davis School of Medicine, Sacramento, CA 95817, USA; jwriess@health.ucdavis.edu (J.W.R.)
* Correspondence: mjttu@health.ucdavis.edu (M.-J.T.) or aimyu@health.ucdavis.edu (A.-M.Y.)
† These authors contributed equally to this work.

Supplementary Table S1-S3

Supplementary Figure S1-S2

Supplemental Table S1. Primary sequences of individual BioRNA/PD-L1-siRNA molecules. Underlined sequences represent the tRNA scaffold. Target siRNA sequences are highlighted in red, and complementary sequences are marked in green. The siRNA antisense sequences were designed to target three different sites within the coding sequence (CDS) and two sites within the 3'UTR of the transcript (NM_014143.4) of the human *CD274* mRNA.

BioRNA	Length (nt)	Sequence (5' to 3')	Target region on *CD274* mRNA	MW (Da)
PD-L1-siRNA-1	180	GCAUGGGUGGUUCAGUGGUAGAAUUCUCGCCUGGCCAGCUGUGAG<br>UGUUUCUUAAUGCGUUCAGCAAAUGCCAGUUGUGAGCAAUAGUAAG<br>GAAGCUGGCAUUGCUAGAACGCAUCUAGAAGUGCUGCACGUUGUUG<br>GCCCACGCGGGAGGCCCGGGUUCGAUUCCCGGCCCAUGCACCA	104-125 (CDS)	58,266
PD-L1-siRNA-2	180	GCAUGGGUGGUUCAGUGGUAGAAUUCUCGCCUGGCCAGCUGUGAGU<br>GUUUCUUUCUGUGAUCUGAAGUGCAGCAUUGUGAGCAAUAGUAAGG<br>AAGUGCUGCACUCAUGAUCACAGCUAGAAGUGCUGCACGUUGUUGG<br>CCCACGCGGGAGGCCCGGGUUCGAUUCCCGGCCCAUGCACCA	356-377 (CDS)	58,220
PD-L1-siRNA-3	180	GCAUGGGUGGUUCAGUGGUAGAAUUCUCGCCUGGCCAGCUGUGAG<br>UGUUUCUUGUAUGGGGCAUUGACUUUCACAUGUGAGCAAUAGUAAG<br>GAAUGUGGAAGUAAUAGUCCCAUAAUAGAAGUGCUGCACGUUGUUG<br>GCCCACGCGGGAGGCCCGGGUUCGAUUCCCGGCCCAUGCACCA	450-471 (CDS)	58,293
PD-L1-siRNA-4	180	GCAUGGGUGGUUCAGUGGUAGAAUUCUCGCCUGGCCAGCUGUGAG<br>UGUUUCUUAGACUCAAAAUAAAUAGGAAAAUGUGAGCAAUAGUAAGG<br>AAUUUUCCUAUUAUAUUUGAGUCCUAGAAGUGCUGCACGUUGUUGG<br>CCCACGCGGGAGGCCCGGGUUCGAUUCCCGGCCCAUGCACCA	1394-1415 (3'UTR)	58,175
PD-L1-siRNA-5	180	GCAUGGGUGGUUCAGUGGUAGAAUUCUCGCCUGGCCAGCUGUGAG<br>UGUUUCUUGUUCCAUUUUCAGUGCUUGGGCUGUGAGCAAUAGUAAG<br>GAAGCCCAAGCAUGACAAGUGGAAAUAGAAGUGCUGCACGUUGUUG<br>GCCCACGCGGGAGGCCCGGGUUCGAUUCCCGGCCCAUGCACCA	1069-1090 (3'UTR)	58,283

Supplemental Table S2. The sequences of primers utilized for the construction of BioRNA expression plasmids. F, forward; R, reverse.

BioRNA		Cloning Primers (5' to 3')
PD-L1-siRNA-2	F	TTGTAACGCTGAATTCGCATGGGTGGTTCAGTGGTAGAATTCTCGCCTGGCCAGCTGTGAGTGTTTCTTTCTGTGATCTGAAGTGCAGCATTGTGAGCAATAGTAAGGAAGTGCTGCACT
	R	CTTTCGCTAAGGATCTGCAGTGGTGCATGGGCCGGGAATCGAACCCGGGCCTCCCGCGTGGGCCAACAACGTGCAGCACTTCTAGCTGTGATCATGAGTGCAGCACTTCCTT
PD-L1-siRNA-3	F	TTGTAACGCTGAATTCGCATGGGTGGTTCAGTGGTAGAATTCTCGCCTGGCCAGCTGTGAGTGTTTCTTGTATGGGGCATTGACTTTCACATGTGAGCAATAGTAAGGAATGTGGAAGTA
	R	CTTTCGCTAAGGATCTGCAGTGGTGCATGGGCCGGGAATCGAACCCGGGCCTCCCGCGTGGGCCAACAACGTGCAGCACTTCTATTATGGGACTATTACTTCCACATTCCTT
PD-L1-siRNA-4	F	TTGTAACGCTGAATTCGCATGGGTGGTTCAGTGGTAGAATTCTCGCCTGGCCAGCTGTGAGTGTTTCTTAGACTCAAAATAAATAGGAAAATGTGAGCAATAGTAAGGAATTTTCCTAT
	R	CTTTCGCTAAGGATCTGCAGTGGTGCATGGGCCGGGAATCGAACCCGGGCCTCCCGCGTGGGCCAACAACGTGCAGCACTTCTAGGACTCAAATATAATAGGAAAATTCCTT
PD-L1-siRNA-5	F	TTGTAACGCTGAATTCGCATGGGTGGTTCAGTGGTAGAATTCTCGCCTGGCCAGCTGTGAGTGTTTCTTGTTCCATTTTCAGTGCTTGGGCTGTGAGCAATAGTAAGGAAGCCCAAGCAT
	R	CTTTCGCTAAGGATCTGCAGTGGTGCATGGGCCGGGAATCGAACCCGGGCCTCCCGCGTGGGCCAACAACGTGCAGCACTTCTATTTCCACTTGTCATGCTTGGGCTTCCTT

Supplemental Table S3. The sequences of primers used for RT-qPCR analyses in this study.

**Target**		**Primer Sequence (5’-3’)**
PD-L1 siRNA-1	Stem loop RT	GTCGTATCCAGTGCAGGGTCCGAGGTATTCGCACTGGATACGACACTGGC
	Forward	CGCGCAATGCGTTCAGCAAAT
	Reverse	GTGCAGGGTCCGAGGT
U6	Forward	CTCGCTTCGGCAGCACA
	Reverse	AACGCTTCACGAATTTGCGT
IL-2	Forward	AAGAATCCCAAACTCACCAG
	Reverse	CGTTGATATTGCTGATTAAGTCC
IFN-γ	Forward	TCGGTAACTGACTTGAATGTCCA
	Reverse	TCGCTTCCCTGTTTTAGCTGC
Granzyme B	Forward	TGGGGGACCCAGAGATTAAAA
	Reverse	TTTCGTCCATAGGAGACAATGC
PD-L1	Forward	TGCCGACTACAAGCGAATTACTG
	Reverse	CTGCTTGTCCAGATGACTTCGG
18S	Forward	AAGTCCCTGCCCTTTGTACACA
	Reverse	GATCCGAGGGCCTCACTAAAC

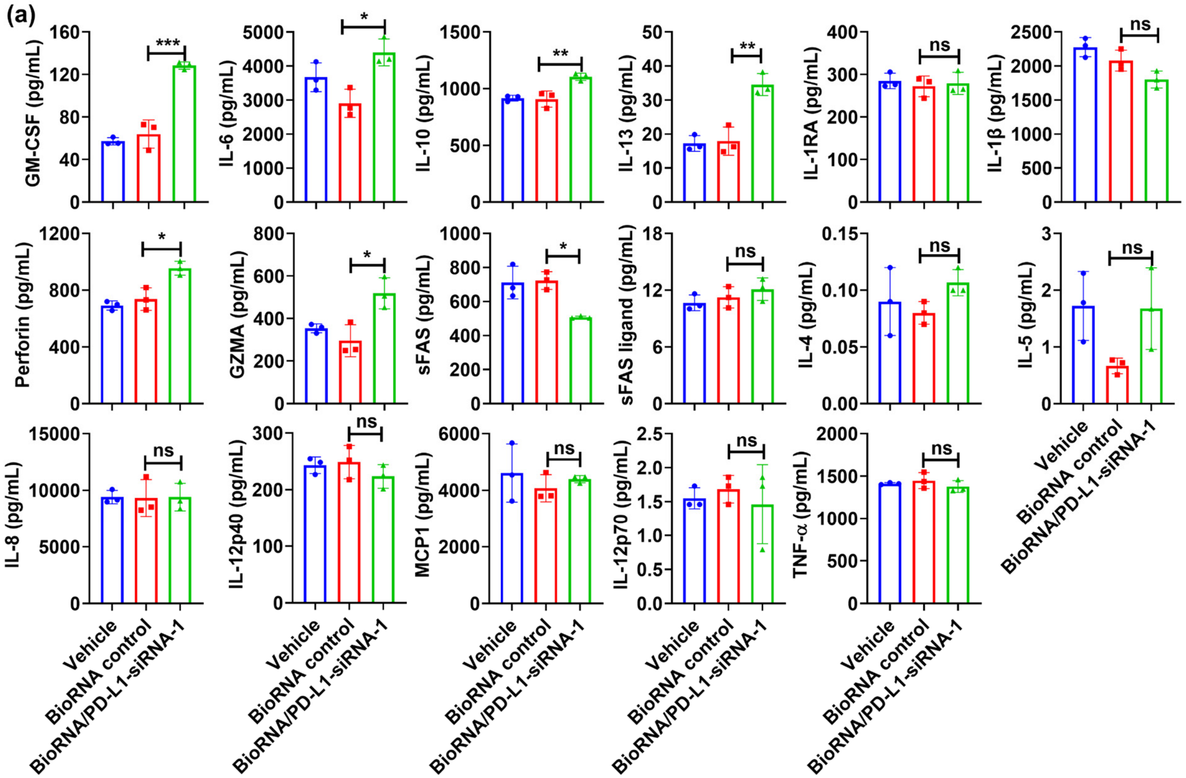
(a)
GM-CSF (pg/mL)
IL-6 (pg/mL)
IL-10 (pg/mL)
IL-13 (pg/mL)
IL-1RA (pg/mL)
IL-1β (pg/mL)
Perforin (pg/mL)
GZMA (pg/mL)
sFAS (pg/mL)
sFAS ligand (pg/mL)
IL-4 (pg/mL)
IL-5 (pg/mL)
IL-8 (pg/mL)
IL-12p40 (pg/mL)
MCP1 (pg/mL)
IL-12p70 (pg/mL)
TNF-α (pg/mL)
***
*
**
**
ns
ns
*
*
*
ns
ns
ns
ns
ns
ns
ns
ns
Vehicle
BioRNA control
BioRNA/PD-L1-siRNA-1

(b)

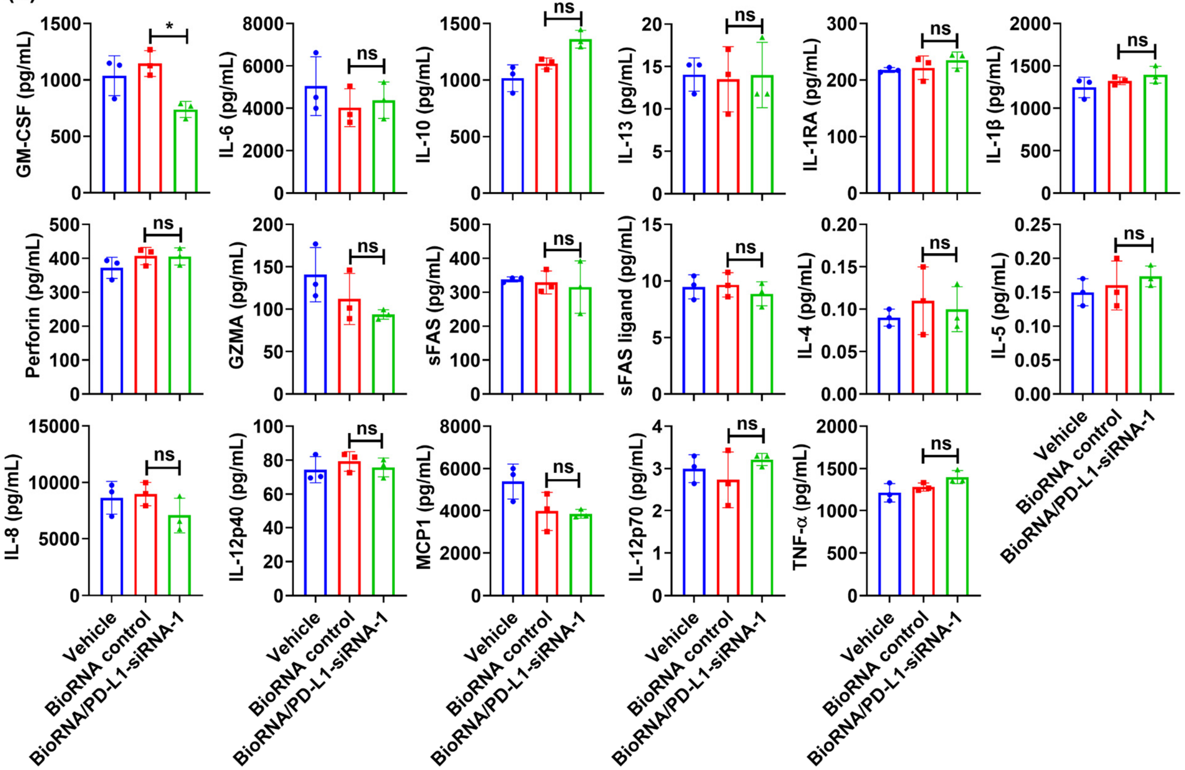

Supplemental Figure S1. Impact of BioRNA/PD-L1-siRNA on the levels of a panel of cytokines in a human NSCLC carcinoma cell-PBMC co-culture system. Different cytokine levels in the cell culture media were measured after co-culture of PBMCs with H460 (**a**) and H1975 (**b**) cells in response to BioRNA/PD-L1-siRNA or control treatment, as determined by Multiplexing LASER Bead Technology. NSCLC cells were treated with 15 nM of RNAs or vehicle for 24 h, and then PBMCs were added to co-incubate with the cancer cells for 48 h. Values are the mean ± SD (n = 3/group). *$P < 0.05$, **$P < 0.01$, ***$P < 0.001$, and ns indicating not significant (one-way ANOVA with Bonferroni post hoc tests).

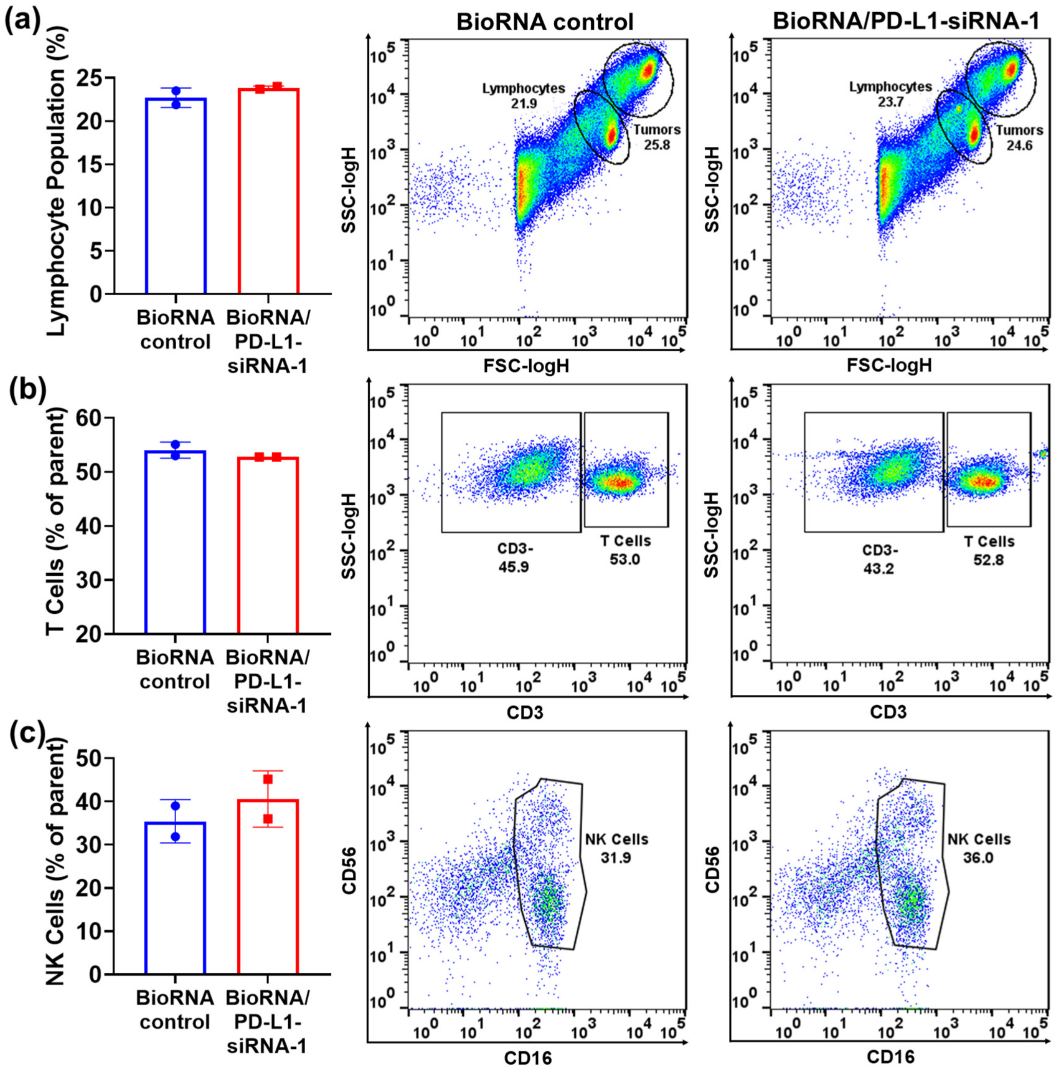

Supplemental Figure S2. Impact of BioRNA/PD-L1-siRNA on the levels of lymphocytes in the human NSCLC cell-PBMC co-culture system. (**a**) Total Lymphocyte, (**b**) T cell, and (**c**) NK cell populations among the H1975 cell-PBMC co-culture system in response to BioRNA/PD-L1-siRNA or control RNA, as evaluated by flow cytometry analysis. Values are the mean ± SD (n = 2/group).
